# SUPPORT: 48-week results of fosamprenavir/ritonavir vs efavirenz with abacavir/lamivudine in under-represented, antiretroviral-naïve patients

**DOI:** 10.1186/1758-2652-13-S4-P7

**Published:** 2010-11-08

**Authors:** P Kumar, E DeJesus, G Huhn, L Sloan, F Garcia, C Small, H Edelstein, F Felizarta, R Hao, B Ha, B Stancil, L Ross, K Oie, K Pappa

**Affiliations:** 1 Georgetown University, Washington DC, USA; 2 Orlando Immunology, Florida, USA; 3 Ruth M. Rothstein CORE Center, Illinois, USA; 4 North Texas Infectious Disease Consultants, Texas, USA; 5 Valley AIDS Council, Texas, USA; 6 New York Medical College, New York, USA; 7 Alameda County Medical Center, California, USA; 8 Private Practice, California, USA; 9 Chase Brexton Health Services, USA; 10 GlaxoSmithKline, RTP, NC, USA

## Purpose of study

The objective of this study was to evaluate the efficacy, tolerability, and safety of fosamprenavir/ritonavir (FPV/r) versus efavirenz (EFV), both in combination with abacavir/lamivudine (ABC/3TC), in a population that is often underrepresented in U.S. clinical trials.

## Methods

In this ongoing 96-week, open-label, prospective, randomized, multicenter study, we compared once-daily ABC/3TC 600 mg/300 mg with FPV 1400 mg/r 100 mg or EFV 600 mg in ARV-naïve, HIV-1-infected subjects with entry viral load (VL) >5,000 c/mL, were HLA-B*5701 negative, and did not have major resistance mutations to study drugs. The primary endpoint was time to switch of third drug or time to development of any treatment-related Grade 3 or 4 adverse events (AEs). Results from the planned 48-week analysis are reported.

## Summary of results

SUPPORT enrolled 32% (32/101) women and 79% (80/101) non-Caucasians. Baseline and demographic characteristics were generally similar between groups. A total of 84 subjects (83%) completed study through W48. Eight patients met the primary endpoint: 3 (6%) and 5 (10%) on FPV/r and EFV, respectively. At W48, by ITT-Exposed missing-equals-failure analysis, 76% (39/51) and 82% (41/50) of subjects achieved VL <50 c/mL on FPV/r vs. EFV, respectively. Median change from baseline to W48 in CD4 cell count was 178 cells/mm^3^ in each group. Rate of treatment-related grade 2-4 AEs was lower in the FPV/r-arm (9/51, 18%) vs. the EFV-arm (15/50, 30%) primarily due to EFV-related rash and dizziness (8% each). Rates of treatment-related serious AEs and grade 3-4 lab abnormalities were similar between FPV/r vs. EFV. A total of 8 virologic failures occurred through W48. At failure, HIV PRO or RT treatment-emergent mutations were present in 4 of 5 EFV patients and 1 of 3 FPV/r patients selected an RT mutation. Median change from BL in total/HDL cholesterol ratio was unchanged in both groups but the FPV/r arm had larger changes in triglycerides (32 vs. 7 mg/dL) and in LDL cholesterol (22 vs. 11 mg/dL).

## Conclusions

Through 48 weeks, in a diverse population, virologic/immunologic responses were not demonstrably different between FPV/r and EFV when given with ABC/3TC, but the EFV regimen had slightly more patients meeting the tolerability endpoint, treatment-related grade 2-4 AEs, virologic failures, and treatment-emergent mutations at failure.

